# Bladder Stone Formation Due to Suture Migration After Burch Colposuspension: A Case Report

**DOI:** 10.7759/cureus.101020

**Published:** 2026-01-07

**Authors:** Berk Y Ekenci, Abdul S Erkartal, Alihan Kokurcan, Hilmi Sari, Ahmet N Karakoyunlu

**Affiliations:** 1 Urology, Kastamonu Inebolu State Hospital, Kastamonu, TUR; 2 Urology, Ankara Etlik City Hospital, Ankara, TUR; 3 Urology, Hitit University Çorum Erol Olçok Training and Research Hospital, Çorum, TUR

**Keywords:** burch colposuspension, postoperative complications, sutures, urinary bladder calculi, urinary stress incontinence

## Abstract

Burch colposuspension is a well-established, mesh-free surgical technique for the treatment of stress urinary incontinence. Although lower urinary tract injuries may occur during the procedure, complications related to suture migration into the bladder are rare and may present years after surgery. We report a case of a 50-year-old woman who presented with a one-year history of progressive dysuria, suprapubic pain, and dyspareunia. She had undergone laparoscopic Burch colposuspension eight years earlier. Ultrasonography and computed tomography revealed bladder stones, and urethrocystoscopy demonstrated migrated polypropylene suture material within the bladder lumen serving as a nidus for stone formation. Endoscopic holmium laser lithotripsy was performed, and both the stones and suture materials were successfully removed. The patient’s symptoms resolved completely during follow-up. Suture migration following Burch colposuspension should be considered in patients presenting with chronic urinary symptoms and a history of incontinence surgery, regardless of the time elapsed since the procedure.

## Introduction

Burch colposuspension is a well-established, mesh-free surgical technique for the treatment of stress urinary incontinence and is based on suspending the paraurethral vaginal wall to the Cooper (iliopectineal) ligament using non-absorbable polypropylene sutures. Although the paraurethral vaginal tissue and bladder are usually clearly distinguishable during surgery, inadvertent passage of sutures through the bladder wall may rarely occur, resulting in intravesical foreign body reactions and subsequent urinary symptoms [1].

Lower urinary tract injuries have been reported in up to 9.6% of colposuspension procedures, whereas complications specifically related to non-absorbable suture material are considerably less common [2]. Suture-related complications may present as early or late migration into the bladder lumen. Late migration is generally attributed to chronic inflammatory processes leading to gradual erosion of the suture through the bladder wall [1,3]. Once exposed to the bladder lumen, suture material may act as a nidus for crystallization and subsequent bladder stone formation, even in the absence of infection or metabolic abnormalities [4].

Reports of suture erosion following Burch colposuspension remain limited, with the interval between surgery and diagnosis ranging from several months to more than 20 years [1,5-8]. Due to the prolonged latency period and non-specific clinical presentation, diagnosis may be delayed unless a history of prior incontinence surgery is carefully considered. In this study, we describe the endoscopic management of migrated suture material associated with bladder stone formation in a patient who presented with chronic pelvic pain eight years after laparoscopic Burch colposuspension.

## Case presentation

A 50-year-old woman with a body mass index of 25.9 kg/m² presented with a one-year history of progressively worsening dysuria, suprapubic pain, and dyspareunia. Her medical history revealed that she had undergone laparoscopic Burch colposuspension eight years earlier for stress urinary incontinence.

**Figure 1:**
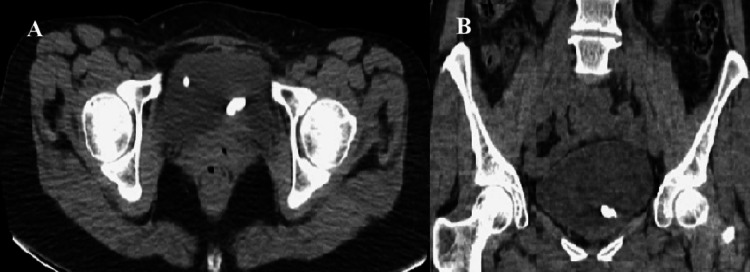
Computed tomography image demonstrating bladder stones. (A) Axial section and (B) coronal section.

Physical examination was unremarkable. Urinalysis demonstrated 42 erythrocytes and five leukocytes per high-power field, while urine culture showed no bacterial growth. Initial ultrasonography followed by computed tomography of the urinary system revealed two bladder stones, the larger measuring approximately 1 cm in diameter (Figures 1A, 1B).

To exclude a gynecological cause of her symptoms, a gynecological examination was performed under general anesthesia due to significant pelvic pain, and no pathological findings were identified. Subsequent urethrocystoscopy demonstrated a 5-mm stone overlying polypropylene suture material at the junction of the right lateral and anterior bladder wall at the 10 o’clock position, as well as an approximately 1-cm stone covering suture material on the left lateral wall at the 3 o’clock position.

**Figure 2:**
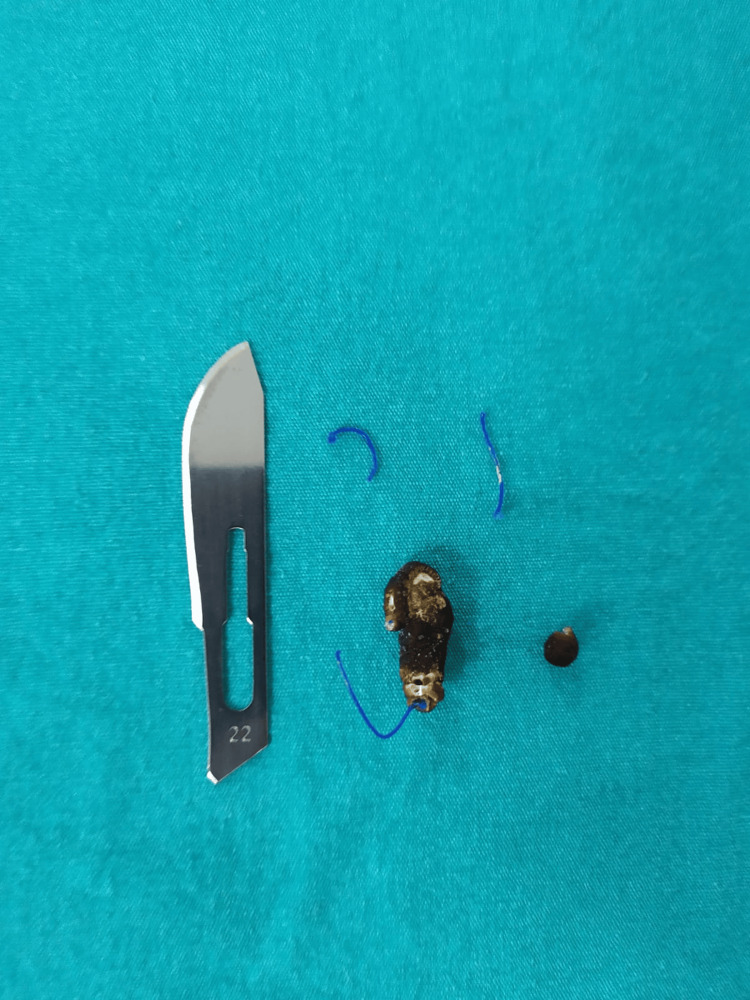
Removed bladder stone and polypropylene suture materials. A surgical blade is shown for size reference.

Holmium laser lithotripsy (2 J/6 Hz) was applied to the stones overlying the sutures, and both the stone fragments and suture materials were subsequently removed endoscopically using forceps (Figure 2). The urethral catheter was removed on postoperative day one, and the patient was discharged. At postoperative follow-up visits at one, three, and six months, her symptoms gradually improved and ultimately resolved completely.

## Discussion

Intravesical migration of suture material following Burch colposuspension represents an uncommon but clinically relevant complication that may manifest long after the initial surgical intervention. Owing to its delayed onset and non-specific symptom profile, diagnosis is often postponed until secondary complications such as bladder stone formation occur. The present case highlights the importance of maintaining clinical suspicion for suture-related pathology in patients with a history of incontinence surgery who present with chronic or progressive urinary symptoms.

Previous reports indicate that both open and laparoscopic colposuspension procedures may be complicated by intravesical foreign material, most commonly identified during evaluation for hematuria, irritative voiding symptoms, or chronic pelvic pain [1,5-8]. The wide variation in the reported interval between surgery and symptom onset suggests that the underlying pathophysiological process is frequently indolent, allowing encrustation and stone formation to progress silently over time. This delayed clinical presentation may further contribute to underrecognition of the condition.

Imaging modalities, such as ultrasonography and computed tomography, play an important role in the initial detection of bladder stones; however, they are insufficient to determine the underlying etiology. Definitive diagnosis relies on direct endoscopic visualization, which enables simultaneous identification of both the bladder stone and the causative suture material, as well as assessment of the depth of suture penetration into the bladder wall [5,6].

Endoscopic management is widely regarded as the preferred treatment modality for intravesical suture-related complications, offering a minimally invasive approach with favorable outcomes [1,5-7]. Holmium laser lithotripsy combined with endoscopic removal of the suture material has demonstrated high success rates, particularly in cases with limited stone burden and superficial suture erosion. Nevertheless, treatment strategies should be individualized based on stone size, degree of encrustation, and the extent of suture embedding within the bladder wall.

Although endoscopic techniques are effective in the majority of cases, open surgical approaches such as cystotomy or cystolithotomy have been reported in selected patients when endoscopic removal is unsuccessful or technically unfeasible due to extensive encrustation or deeply embedded suture material [9]. Similar open surgical interventions have also been described following other incontinence procedures when intravesical foreign material could not be safely removed endoscopically [10,11]. These findings indicate that while endoscopic treatment remains the first-line approach, open surgery continues to play a role in the management of complex cases. In contrast, complete endoscopic removal was successfully achieved in our patient, resulting in symptom resolution without the need for open intervention.

Over the past decade, advances in surgical techniques and technology have led to widespread adoption of vaginal mesh procedures due to satisfactory clinical outcomes and a relatively short learning curve, contributing to a decline in the use of the Burch procedure [12]. However, in 2019, the U.S. Food and Drug Administration (FDA) banned the manufacture and sale of transvaginal mesh products because of safety concerns related to complications. Following this decision, renewed interest in mesh-free surgical approaches has been observed, and Burch colposuspension has regained popularity among surgeons [13]. As a consequence, an increase in long-term suture-related complications similar to those observed in our case may be anticipated in the coming years.

## Conclusions

When evaluating patients with chronic urinary symptoms, a history of prior incontinence surgery should be carefully assessed. Regardless of the time elapsed since surgery, the possibility of bladder stone formation due to suture migration should be considered in the differential diagnosis. In addition, postoperative urethrocystoscopy may play an important role in the early detection of suture migration, particularly in patients presenting with persistent or recurrent urinary symptoms following incontinence surgery.
